# No significant relationship exists between tumor size and prognosis in distant metastatic hepatocellular carcinoma: a propensity score matching analysis based on SEER database

**DOI:** 10.1186/s12876-022-02355-1

**Published:** 2022-06-02

**Authors:** Jun Xie, Chunyao Zheng, Jinliang Xie, Fangfei Wang, Dingwei Liu, Rong Zeng, Chensong Yu, Sihai Chen

**Affiliations:** 1 Department of Gastroenterology, The First Affiliated Hospital of Nanchang University, No. 17 Yongwaizheng Street, Nanchang, Jiangxi Province China; 2 Gastroenterology Institute of Jiangxi Province, Nanchang, Jiangxi Province China; 3 Nanchang University, Nanchang, China

**Keywords:** Tumor sizes, Distant metastatic hepatocellular carcinoma, Propensity score matching, Prognosis

## Abstract

**Background:**

Previous studies have shown that tumor size has an impact on the prognosis of hepatocellular carcinoma (HCC). Whether tumor size is related to the prognosis of distant metastatic HCC is unclear. The purpose of this study was to investigate the effect of tumor size on the prognosis of distant metastatic HCC.

**Methods:**

Data on patients with HCC were collected from the (SEER) database of surveillance, epidemiology and final results. Propensity score matching (PSM) was used to reduce confounding factors and comprehensively evaluate the clinicopathological features and prognosis of distant metastatic HCC.

**Results:**

There were 189 patients with distant metastatic HCC whose tumor size was ≤ 50 mm and 615 patients with a tumor size > 50 mm. The tumor sizes of distant metastatic HCC patients were associated with race, grade, surgical treatment, N and AFP. The Kaplan–Meier analysis showed that the mortality rate of patients with a tumor size > 50 mm was higher than that of patients with a tumor size ≤ 50 mm (*p* = 0.00062). However, there were no significant differences in mortality rates after adjusting for confounding variables by using propensity score matching (*p* = 0.23).

**Conclusion:**

This propensity score matching study provides the best data in support of the following assertions: tumor size is not an independent prognostic factor for distant metastatic HCC.

**Supplementary Information:**

The online version contains supplementary material available at 10.1186/s12876-022-02355-1.

## Introduction

Hepatocellular carcinoma (HCC) accounts for 75–85% of primary liver cancer (PLC), and is the main histological type of primary liver cancer [1, 2]. HCC ranks third in cancer-related mortality, causing over 500,000 deaths worldwide annually [3]. The prevalence of HCC is significantly higher in North and West Africa, and East and South-East Asia, and 50% of reported HCC cases originate in China. HCC is associated with poor prognosis, and the incidence of HCC is increasing in many countries [4]. Although hepatectomy provides a chance of cure or a prolonged life expectancy, it is only feasible in 20–40% of patients. Other local treatments, such as radiofrequency ablation, transcatheter arterial chemoembolization, ethanol injection and sorafenib, prolong the lives of patients with resectable tumors [5]. Although progress has been made in the diagnosis and treatment of HCC, the median survival time of patients with HCC is 33 months [6], and the prognosis can still be improved. There are many factors affecting the prognosis of HCC, including portal hypertension, the level of bilirubin, the tumor number and vascular invasion [7]. Therefore, it is critical to determine the prognostic factors related to clinical outcomes.

Many studies have shown that tumor size is a poor prognostic factor for various cancers, such as colon cancer [8–11], esophageal cancer [12], breast cancer [13, 14] and thyroid cancer [15]. Therefore, many scholars have explored the relationship between tumor size and HCC patient prognosis. However, whether tumor size can be used as an independent factor to predict the prognosis of hepatocellular carcinoma (HCC) patients is still controversial. Some researchers have suggested that tumor size is a prognostic factor in HCC. In most cases, the larger the tumor is, the worse the prognosis will be. Tumor size has been included in most HCC surgical staging systems [16, 17]. However, several large studies [18, 19] subsequently showed that tumor size is not a single independent prognostic factor in HCC, and large hepatocellular carcinoma is associated with poor prognosis because of its association with other adverse prognostic factors, such as vascular invasion, tumors with higher multifocality and tumors of higher, grades. A recent study showed that tumor size at diagnosis can be used as an independent risk predictor of histological grade, staging, surgical choice and survival outcome in HCC patients [20].

In this study, we collected data on HCC patients from the SEER database from 2010 to 2015, and screened the data of advanced liver cancer patients for analysis. The propensity matching score was used to evaluate whether tumor size could be used as an independent prognostic factor to predict the survival outcome of advanced HCC patients.

## Materials and methods

### Data source

The SEER database is an authoritative cancer statistics database in the United States, that records the morbidity rate, mortality rate and disease characteristics of millions of malignant tumor patients across the United States. The SEER database aims to reduce the cancer burden in the American population. The tumor information in the database is unified and standardized by SEER*Stat software and updated regularly. The data used in this study were all obtained from this database.

### Patient selection process

**Fig. 1:**
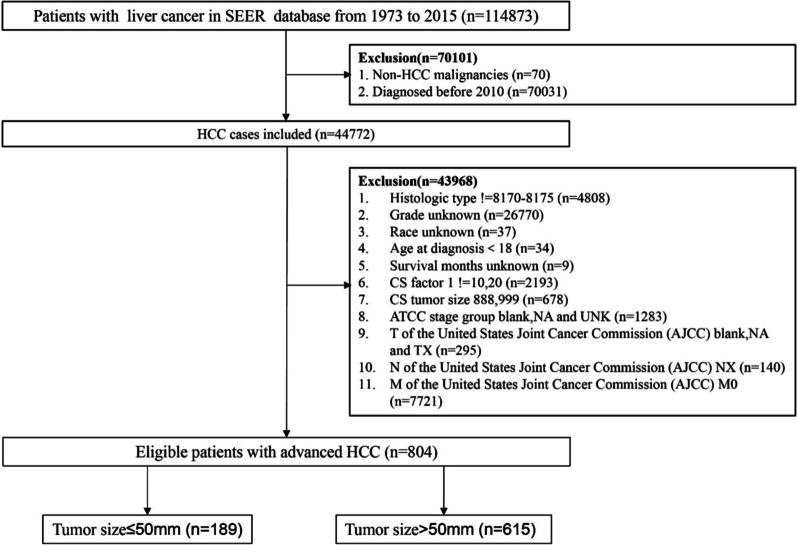
Patient selection process

In this study, we used SEER*Stat software (version 8.3.2) to extract the clinical information of 114,873 patients with liver cancer from 1973 to 2015 from the SEER database. First, a total of 70,101 patients without liver cancer who were diagnosed before 2010 were excluded. Then, we removed patients with liver cancer tissue types that were not hepatocellular carcinoma. In addition, patients with nonadvanced liver cancer whose pathological stages were classified according to the AJCC 7th edition TNM staging system of the American Joint Commission on Cancer were excluded. The additional exclusion criteria included the following: unknown grade information, unknown survival time, unknown tumor size, age at diagnosis < 18, and unknown race information. The selection criteria for patients are shown in (Fig. 1).

### Propensity score matching

In observational studies, propensity score matching (PSM) is a statistical method which used to reduce the influence of these biases and confounding variables, and allow for a more reasonable comparison between the experimental group and the control group [21]. The number of patients with > 50 mm tumors was much larger than the number of patients with ≤ 50 mm tumors. Therefore, the imbalance in the baseline characteristics associated with prognosis may influence the estimation of the prognostic impact of tumor size. To adjust these key covariates, we implemented PSM. In order to prevent the emergence of new confounding factors, all variables were included in PSM. The PSM analysis included 8 unbalanced covariates (Age, Race, Grade, Sex, Surgical treatment, T, N, AFP). PSM uses 1:1 nearest neighbor matching to establish a pair of matches between the experimental group and the control group. Each PSM covariable was estimated using a logistic regression model.

## Statistical analysis

We used SPSS Statistics version 21.0 (SPSS Inc., Chicago, IL) and the R statistical computing environment (R Foundation for Statistical Computing, Vienna, Austria)) for data analysis. Continuous variables are expressed as the mean and standard deviation, and classified data are expressed as counts and percentages, which are used in survival analysis. The MatchIt package was used to perform PSM, propensity scores estimated with logistic regression. The caliper width is set to 0.1. The standardized mean difference method (SMD) was used to compare the balance between the tumor size ≤ 50 mm group and the tumor size > 50 mm group, with a value greater than 0.10 indicating imbalance [22]. The MBESS package was used to calculate the SMD value. Pearson’s chi-square test was used to compare the baseline characteristics and differences between the two groups. Continuous variables were analyzed by a t-test. The multivariate analysis was performed using the Cox proportional hazard model. Cox regression analysis was used to calculate the hazard ratio (HR) and 95% confidence interval (95% CI).

## Results

Clinicopathological characteristics of distant metastatic hepatocellular carcinoma patients.

**Fig. 2:**
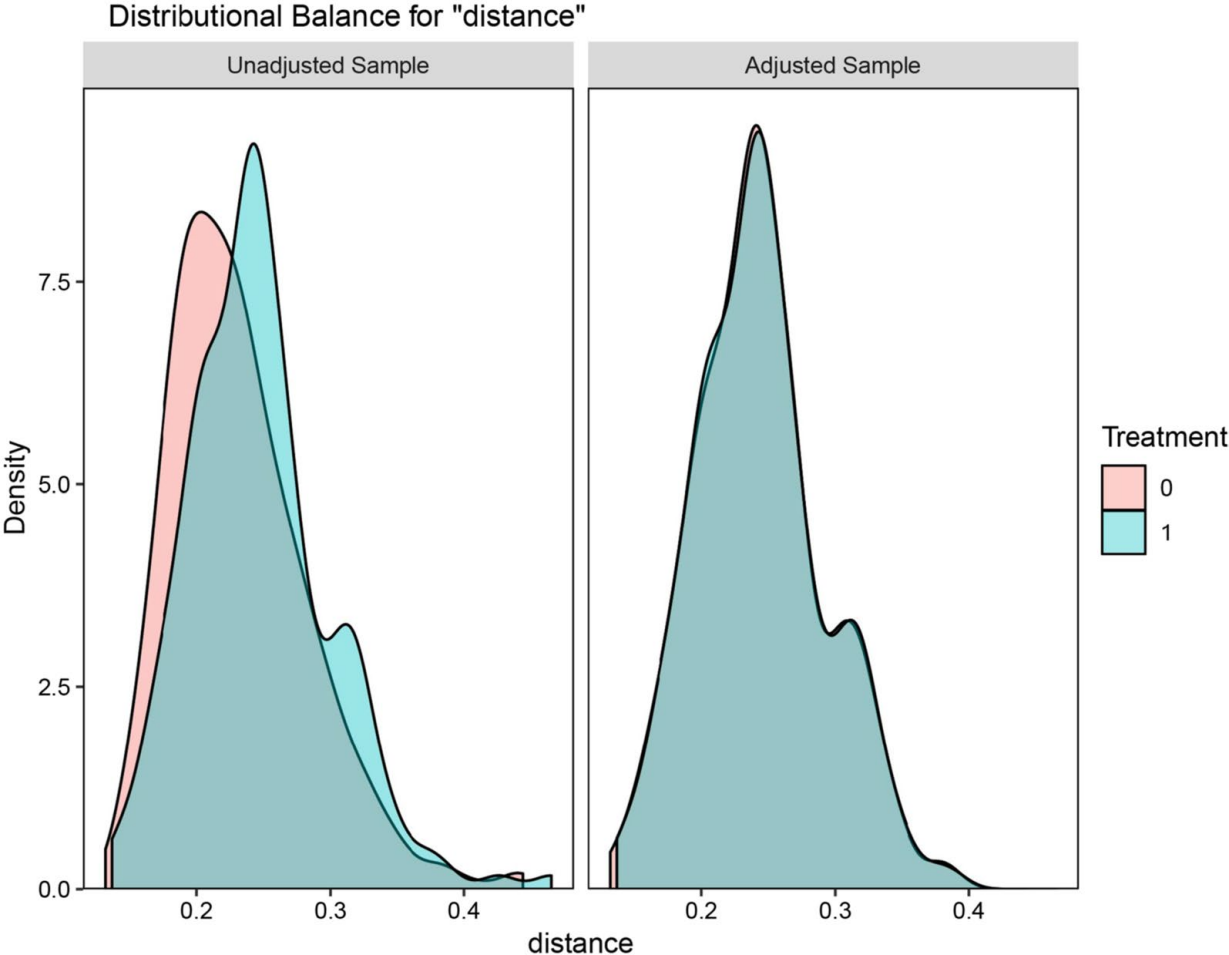
Distributional balance for “distance”

A total of 804 patients with HCC treated from 2010 to 2015 were divided into two groups with 50 mm as the cut-off point for tumor size. The baseline characteristics of 189 patients with tumor size ≤ 50 mm and 615 patients with tumor size > 50 mm are shown in (Table 1). We selected HCC patients with distant metastasis in stage M1. The average age of patients with tumors ≤ 50 mm was 63.4 years, and the average age of patients with tumors > 50 mm was 64.6 years. We observed that regardless of the size of the tumor, most of the patients with advanced hepatocellular carcinoma were male, and most of them were AFP-positive patients who had not undergone surgery. Both before and after propensity score matching, the tumor size of HCC patients was not related to age, sex, race (American Indian / AK Native, Asian / Pacific Islander), grade, surgical treatment, N, or AFP (*p* > 0.05). However, before PSM, the SMD values of race、grade、surgical treatment, N and AFP were all greater than 0.1, indicating that these factors had a certain impact on our analysis. Therefore, the PSM method was used to reduce the influence of confounding factors. The propensity scores of hepatocellular carcinoma patients in different groups in the selected SEER database were matched (Fig. 2). After PSM, all of the SMD values were lower than 0.1 (Table 2), indicating that all baseline variables were matched completely among the 186 selected patients with tumors ≤ 50 mm and the 186 matched patients with tumors > 50 mm.

**Table 1:** Characteristics of the distant metastatic HCC patients before propensity score matching

Characteristic	Before matching			
	Tumor size ≤ 50mm n = 189 (23.5%)	Tumor size > 50mm n = 615 (76.5%)	*P* value	SMD
Age (year)	63.4 ± 11.5	64.6 ± 12.0	0.585	0.094
Race			0.178	0.149
Black	37 (19.6)	89 (14.5)		
White	123 (65.1)	410 (66.7)		
Other	29 (15.3)	116 (18.9)		
Grade			0.088	0.142
Well differentiated	50 (26.5)	117 (19)		
Moderately Differentiated	66 (34.9)	240 (39)		
Poorly differentiated and undifferentiated	73 (38.6)	258 (42)		
Sex			0.666	0.036
Male	153 (81)	489 (79.5)		
Female	36 (19)	126 (20.5)		
Surgical treatment			0.052	0.139
Wedge or segmental resection	16 (8.5)	38 (6.2)		
Local tumor destruction	8 (4.2)	10 (1.6)		
No surgery	165 (87.3)	567 (92.2)		
T			< 0.001	0.657
T0, T1	53 (28)	151 (24.6)		
T2	98 (51.9)	19 (3.1)		
T3	27 (14.3)	367 (59.7)		
T4	11 (5.8)	78 (12.7)		
N			0.423	0.666
N0	134 (70.9)	417 (67.8)		
N1	55 (29.1)	198 (32.2)		
AFP			0.167	0.115
Negative	45 (23.8)	118 (19.2)		
Positive	144 (76.2)	497 (80.8)		
M	All is M1

**Table 2:** Characteristics of the distant metastatic HCC patients after propensity score matching

Characteristic	After matching			
	Tumor size ≤ 50mm n = 186 (50%)	Tumor size > 50mm n = 186 (50%)	*P* value	SMD
Age (year)	63.5 ± 11.6	62.7 ± 12.3	0.586	0.067
Race			0.513	0.038
Black	35 (18.8)	28 (15)		
White	122 (65.6)	132 (71)		
Other	29 (15.6)	26 (14)		
Grade			0.541	0.014
Well differentiated	48 (25.8)	42 (22.6)		
Moderately differentiated	65 (34.9)	75 (40.3)		
Poorly differentiated and undifferentiated	73 (39.3)	69 (37.1)		
Sex			0.895	0.014
Male	150 (80.6)	151 (81.2)		
Female	36 (19.4)	35 (18.8)		
Surgical treatment			0.309	0.049
Wedge or segmental resection	14 (7.5)	14 (7.5)		
Local tumor destruction	8 (4.3)	3 (1.6)		
No surgery	164 (88.2)	169 (90.9)		
T			< 0.001	0.657
T0, T1	52 (28)	48 (25.8)		
T2	97 (52.2)	6 (3.2)		
T3	26 (14)	108 (58.1)		
T4	11 (6)	24 (12.9)		
N			0.731	0.036
N0	131 (79.4)	134 (72)		
N1	55 (20.6)	52 (28)		
AFP			0.543	0.063
Negative	42 (22.6)	47 (25.3)		
Positive	144 (77.4)	139 (74.7)		
M	All is M1			

Effect of tumor size on the overall survival (OS) rate of patients with advanced hepatocellular carcinoma.

**Fig. 3:**
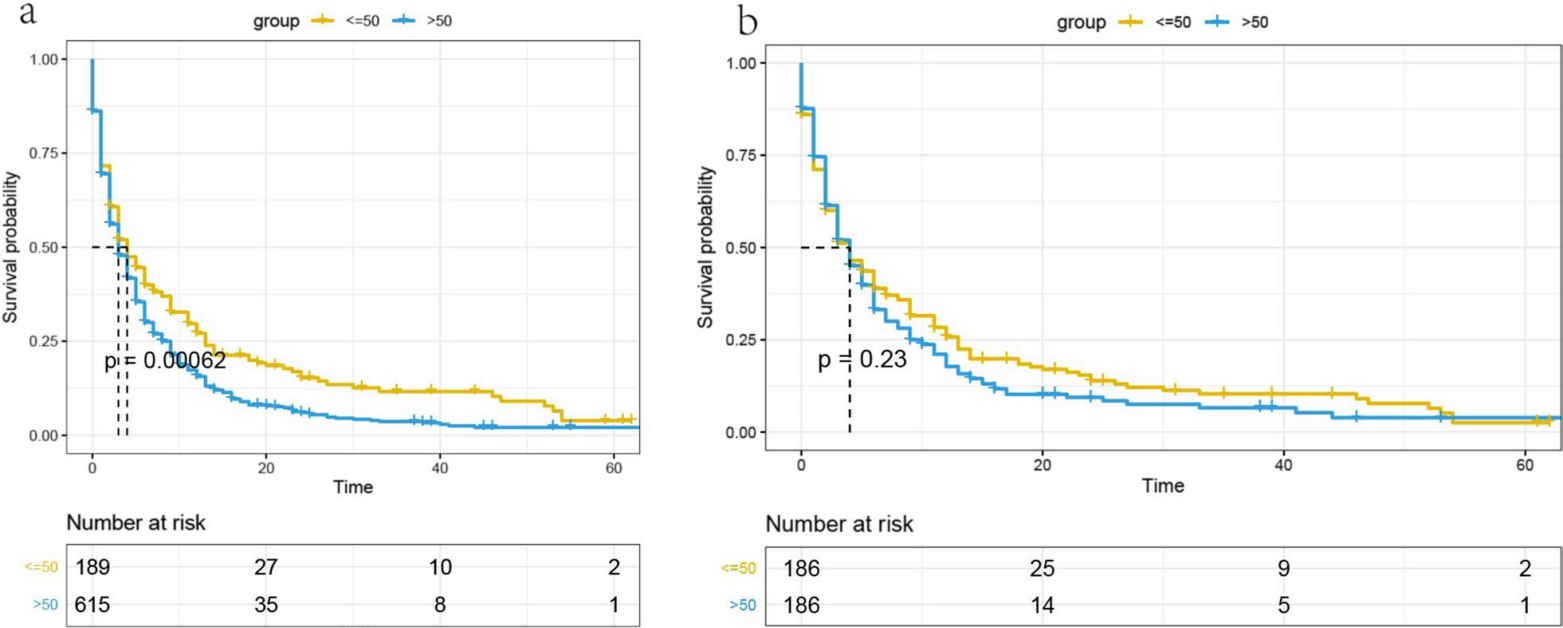
Comparison of overall survival between the tumor size ≤ 50 mm group and the tumor size > 50 mm group before (**a**) and after (**b**) propensity score matching

We performed Kaplan–Meier analysis before and after PSM. The Kaplan–Meier analysis and log-rank test showed that before PSM, the mortality rate of patients with tumor size > 50 mm was significantly higher than that of patients with tumor size ≤ 50 mm (*P* = 0.00062) (Fig. 3a). Propensity score matching was performed to minimize any deviations resulting from the influence of age, race, sex, surgical treatment, tumor grade, and AFP. After matching all potential confounding factors, there was no significant difference in the survival rate of patients with advanced liver cancer with a tumor size > 50 mm and that of those with a tumor size ≤ 50 mm (*P* = 0.23) (Fig. 3b).

## Discussion

In this study, we collected and analyzed the SEER database data on 804 patients with type M1 hepatocellular carcinoma. The results showed that the p values of all the influencing factors were below 0.05, but the distance balance distribution curve revealed an imbalance, thus, we used the SMD value for the analysis. We used PSM to exclude all confounding factors, and we observed that all clinical features were matched between the group with tumor sizes ≤ 50 mm and the group with tumor sizes > 50 mm. In addition, we analyzed the survival rates of the two groups before and after matching. The results showed that tumor size could not independently predict the prognosis of M1 hepatocellular carcinoma patients.

The incidence of HCC increasing worldwide. HCC is an invasive malignant tumor and one of the most common causes of cancer-related death. Surgery is the main choice for the treatment of hepatocellular carcinoma and is, most effective treatment, for obtaining the best overall survival rate and recurrence-free survival rate [23]. A considerable proportion of HCC patients present with advanced large hepatocellular carcinoma at the time of the initial diagnosis. When liver function is maintained within an acceptable range, surgical treatment is considered to be the first choice for these large liver cancers [24]. Radiofrequency ablation (RFA) is the preferred treatment for patients who are not eligible for surgery or orthotopic liver transplantation. Postoperative recurrence is closely related to patient survival [25], and angiotensin receptor blockers can improve the survival outcomes of HCC patients after RFA [26]. However, in our study, the vast majority of advanced HCC patients did not receive surgical treatment, and we speculate that some patients may have died before receiving surgery or that their bodies deteriorated to the extent that they were unable to withstand surgical treatment. We also observed that the survival rate of AFP-positive patients was lower than that of AFP-negative patients. This finding is in agreement with the conclusion of a previous study in which it was shown that AFP level is an independent risk factor associated with tumor differentiation, TNM stage, tumor size and survival outcome among in patients with liver cancer. Compared with AFP-negative tumors, AFP-positive tumors had lower differentiation levels [27, 28], were of later TNM stage [29, 30], and patients had larger tumors and lower survival rates [29, 31–34]. In addition, some comorbidities can also affect the occurrence and prognosis of HCC. For example, due to the prevalence of obesity and type-2 diabetes mellitus, Non Alcoholic Fatty Liver Disease (NAFLD) /is now becoming a major risk factor of HCC, and distant metastasis is more likely to occur in HCC patients complicated with diabetes, thus affecting patient survival [35].

The typical feature of most malignant tumors is growth accompanied by distant organ metastasis, which is the main factor leading to death. Hepatocellular carcinoma most frequently metastasizes to the lungs, followed by the bones and other sites [32–34]. When distant metastasis occurs, adverse reactions in various tissues and organs of the body aggravate the patient's condition and eventually lead to death. Relatively speaking, the size of the tumor may have little effect on the patient. The purpose of this study was to better understand the effect of tumor size on the prognosis of liver cancer patients with distant metastases. Larger tumors are generally associated with poorer survival outcomes, this may make patients with larger tumors more anxious. Emotions have an impact on survival in patients with advanced cancer [36]. This study may reduce anxiety in some patients with large tumor and provide guidance for clinicians in choosing treatment options. Further understanding of the effect of tumor size on the survival rate of patients with distant metastasis will help to correctly classify advanced HCC patients and may provide a reference for individualized and targeted therapy.

Most previous studies focused on the effect of tumor size on the prognosis of patients with hepatocellular carcinoma (HCC). This is the first study of the effect of tumor size on the survival rate of HCC patients with distant metastases. This study also has some shortcomings. First, after performing PSM, the patient' sample size decreased. Second, the SEER HCC database does not include information regarding recurrence; therefore, the relationship between tumor size and recurrence could not be analyzed. Both before and after propensity score matching, the tumor size of HCC patients was not related to age, sex, race (American Indian / AK Native, Asian / Pacific Islander), grade, surgical treatment, N, or AFP (*p* > 0.05). Third, although our study population included white, black, and other (American Indian / AK Native, Asian / Pacific Islander), they were all treated in the United States, so there may exist regional differences. Whether the conclusions of this study are applicable to other regions, such as Europe and Asia, needs further discussion.

## Supplementary Information

**Additional file 1.** Primary data of this study.

**Additional file 2.** **Table S1.** Univariate Cox regression analyses of prognostic factors in patients with distant metastatic HCC after PSM.

## Data Availability

The datasets used and/or analysed during the current study available from the corresponding author on reasonable request. The data used in this study are available free of charge online at http://www.seer.cancer.gov on request.
