# Correction: No significant relationship exists between tumor size and prognosis in distant metastatic hepatocellular carcinoma: a propensity score matching analysis based on SEER database

**DOI:** 10.1186/s12876-022-02472-x

**Published:** 2022-08-25

**Authors:** Jun Xie, Chunyao Zheng, Jinliang Xie, Fangfei Wang, Dingwei Liu, Rong Zeng, Chensong Yu, Sihai Chen

**Affiliations:** 1 Department of Gastroenterology, The First Affiliated Hospital of Nanchang University, No. 17 Yongwaizheng Street, Nanchang, Jiangxi Province China; 2 Gastroenterology Institute of Jiangxi Province, Nanchang, Jiangxi Province China; 3 Nanchang University, Nanchang, China

## Correction to: BMC Gastroenterology (2022) 22:274 10.1186/s12876-022-02355-1

After publication of this article [1], the authors reported that in the Funding section the grant number was incorrectly given as ‘20171BBG70083’ and should have been ‘20212BAG70026’.

The original article [1] has been corrected.
