# Supplementary material for: No significant relationship exists between tumor size and prognosis in distant metastatic hepatocellular carcinoma: a propensity score matching analysis based on SEER database
Source: BMC Gastroenterol. 2022 Jun 2;22:274. doi: 10.1186/s12876-022-02355-1 (PMC9161599; doi:10.1186/s12876-022-02355-1)
Supplement: Supplementary file 2 — Additional file 2. Table S1. Univariate Cox regression analyses of prognostic factors in patients with distant metastatic HCC after PSM. [file 12876_2022_2355_MOESM2_ESM.docx]

SUPPLEMENTARY TABLE 1: Univariate Cox regression analyses of prognostic factors in patients with distant metastatic HCC after PSM

| Characteristic | Hazard ratio | 95％CI | P value |
| --- | --- | --- | --- |
| Group |  |  |  |
| Tumor size≤50mm |  |  |  |
| Tumor size>50mm | 1.220 | 0.978-1.523 | 0.079 |
